# Molecular insights into anti-inflammatory activities of selected Indian herbs

**DOI:** 10.1016/j.jaim.2024.101081

**Published:** 2025-03-27

**Authors:** Saumya Upadhyay, Rajan swami, Shweta Shrivastava, Manish Kumar Jeengar

**Affiliations:** a Department of Pharmacology, Amrita School of Pharmacy, Amrita Vishwa Vidyapeetham, AIMS Health Sciences Campus, Kochi, 682041, Kerala, India; b Chitkara College of Pharmacy, Chitkara University, 140 401, Punjab, India; c School of Pharmacy, School of Health & Allied Sciences, ARKA JAIN University, Gamaharia, Seraikela Kharsawan, 832108, Jharkhand, India

**Keywords:** Inflammation, Traditional indian herbs, Anti-inflammatory mechanisms, Molecular targets, Phytotherapy, Chronic inflammatory disorders

## Abstract

Inflammation is a universal response of mammalian tissue to harm, comprising reactions to injuries, pathogens, and foreign particles. Chronic inflammation, often present in allergies and autoimmune disorders, poses significant risks, potentially leading to conditions such as rheumatoid arthritis, Alzheimer's disease, asthma, and inflammatory bowel disease. It can also be a common precursor to cancer. However, Contemporary therapies like NSAIDs and corticosteroids often provide incomplete relief from chronic inflammation and carry significant side effects, underscoring the need for exploring traditional and plant-based medicines for new, effective treatments. As such, there is a growing demand for natural bioactive substances for health maintenance and disease risk reduction. Traditional and plant-based medicines, long-used in managing inflammation and other disorders, hold promise for the discovery of bioactive lead compounds and subsequent drug development for treating inflammatory disorders. This review encompasses an extensive study of the anti-inflammatory potential of selected traditional Indian herbal medicines and the associated pharmacological mechanisms of action. The inflammatory process often entails the activation of transcription factors, induction of various signaling cascades, gene expression, activation of inflammatory enzymes, and release of pro-inflammatory cytokines in inflammatory or immune cells. Detailed exploration of active components in traditional herbal medicines such as the Neem (*Azadirachta indica*), Salai guggul (*Boswellia serrata*), Green tea (*Camellia sinensis*), Saffron (*Crocus sativus*), Turmeric (*Curcuma longa*), Mangosteen (*Garcinia mangostana*), Indian mulberry (*Morinda citrifolia*), Black cumin (*Nigella sativa*), Ashwagandha (*Withania somnifera*), and Ginger (*Zingiber officinale*) reveals their potential anti-inflammatory properties. The in-depth study of these plants provides insight into their potential applications in managing inflammatory disorders. Further research and development are necessary to substantiate these findings and translate them into clinically effective therapeutics.

## 1 Introduction

Inflammation, a multifaceted bodily response to harm, is managed through various therapeutic options, including steroidal, nonsteroidal, and immunosuppressant drugs [1]. Despite their widespread usage, recent years have witnessed a growing shift towards natural remedies and herbal medicines in the treatment of a multitude of diseases. Traditional plant-based medicinal practices are now utilized by a significant portion of the global population, reinforcing their relevance in contemporary healthcare [2].

Medicinal plants have been an integral part of the human health toolkit for centuries, particularly in managing inflammatory diseases such as rheumatoid arthritis, Alzheimer's disease, asthma, inflammatory bowel disease, and systemic lupus erythematosus [3]. Many commonly used traditional herbs have been evaluated for their health benefits in both clinical and experimental settings. These include the Neem tree (*Azadirachta indica*), Salai guggul (*Boswellia serrata*), and Green tea (*Camellia sinensis*). Other notable herbs such as Saffron (*Crocus sativus*), Turmeric (*Curcuma longa*), and Mangosteen (*Garcinia mangostana*) also contribute to this list. Additionally, Indian mulberry (*Morinda citrifolia*), Black cumin (*Nigella sativa*), Ashwagandha (*Withania somnifera*), and Ginger (*Zingiber officinale*) are recognized for their medicinal properties.

These herbs have demonstrated effectiveness in mitigating undesired inflammatory responses, exhibiting considerable anti-inflammatory activity [4]. Phytoconstituents derived from these medicinal plants have gained popularity due to their potency, affordability, and comparatively minimal side effects. They often outperform their synthetic counterparts in terms of efficacy, making them viable candidates for further exploration [5].

This review aims to explore the anti-inflammatory properties, pharmacological mechanisms of action, and potential applications of these selected traditional Indian herbs in treating inflammatory disorders.

## 2 Search strategy

This review entailed a comprehensive search of scientific literature to analyze research on the anti-inflammatory activities of selected traditional Indian herbs, focusing specifically on insights into the molecular pathways and targets involved in these effects. Articles were sourced from Google Scholar, PubMed, and Scopus, with the search being limited to English-language, full-text publications from the period of 2000 to May 2023. The search strategy was designed to identify *in vitro*, and *in vivo* preclinical studies, and clinical evidence evaluating the anti-inflammatory mechanisms of the selected Indian herbs. To achieve this, the search terms included the names of each herb, combined with terms related to anti-inflammatory effects, molecular targets, and pathways. In Supplementary Table 1, we present the specific search terms used for each traditional Indian herb examined in our study. This table outlines our systematic search strategy across major scientific databases, facilitating a thorough investigation into the anti-inflammatory effects and mechanisms of these herbs. The inclusion criteria for this review were: studies investigating the anti-inflammatory activity of the selected Indian herbs; research reporting on the mechanistic anti-inflammatory pathways and targets of these herbs; *in vitro*, *in vivo*, and clinical studies; articles published in English; full-text articles; and studies published from the year 2000 to May 2023. Articles that did not meet these criteria were excluded. Additional exclusion criteria included articles not available in full text or not written in English. Relevant papers that met the criteria for this review article were selected, and two authors independently extracted detailed information regarding each specific herbal remedy, the experimental models used (*in vitro*, *in vivo*, clinical), observed anti-inflammatory effects, and identified molecular targets and pathways. This thorough process ensured the accuracy and reliability of the information presented. This review also provides an extensive discourse on the mechanism of inflammation and the various signaling pathways involved, offering a broader context for understanding the anti-inflammatory activities of the selected traditional Indian herbs.

## 3 Physiology of inflammation

Inflammation is a set of immunological responses characterized by redness, swelling, pain, and heat. The host body produces a defense mechanism to overcome or survive a potentially fatal injury, or infection, and maintain tissue microenvironment in hostile conditions [4,6]. An inflammatory response is categorized into two main classes: acute and chronic inflammation. Acute and chronic inflammation can originate from the several molecular pathways of inflammation which also pave the way for several chronic disorders. A persistent chronic inflammation causes organ dysfunction and death. The mediators released and target tissue affected, differ depending on the type of trauma, injury, and invading organisms [7]. Insights into the role of chemical mediators in inflammation are fundamental. These cell-derived mediators namely histamines, bradykinin, cytokines, chemokines, leukotrienes, nitric oxide, and prostaglandins, also referred to as pro-inflammatory factors, help bring about inflammation and determine the intensity and duration of inflammation. They are either released as plasma proteins, or produced by various cell types, including mast cells, neutrophils, monocytes/macrophages, and platelets, each playing distinct roles in the inflammatory process. These mediators then bind to target receptors to mediate a reaction [1]. There are three principal steps in the inflammation pathway starting with tissue injury, followed by the physiological response (acute inflammatory state), and finally, pathological consequences (chronic inflammatory condition) [6].

### 3.1 Arachidonic acid pathway

Damage to the cells causes the release of phospholipids from the cell membrane's lipid bilayer. With the help of the enzyme phospholipase A2, activated by pro-inflammatory cytokines, phospholipids are degraded to form arachidonic acid. Arachidonic acid is conditionally an essential fatty acid, denoting it comes from linoleic acid which is an essential fatty acid, if there is a linoleic acid deficiency in the body, it must be supplemented through the diet [8]. Arachidonic acid when metabolized with lipoxygenase (LOX) forms leukotrienes (leukotriene B4, C4, D4, and E4). Leukotriene B4 is a chemotaxis agent causing neutrophil extravasation. Leukotriene C4, D4, and E4 are bronchoconstrictors. Another enzyme called cyclooxygenase involved in the inflammatory cascade has two forms cyclooxygenase 1 (COX 1) and cyclooxygenase 2 (COX 2). COX 1 can be nearly found in all tissue whereas COX 2 is prevalent at the inflammatory site. Arachidonic acid when processed with cyclooxygenase forms prostaglandin G2 (PGG2) which then converts to prostaglandin H2 (PGH2) by oxygenation. Depending on the type of tissue, PGH2 in the platelets synthesizes thromboxane A2 (bronchoconstrictor) via thromboxane A2 synthase, carries out thrombosis, and promotes vasoconstriction and platelet aggregation. In the endothelium PGH2 converted to prostacyclin via prostacyclin synthase causes vasodilation and leaves the blood flowing smoothly through the endothelium, but inhibits platelet aggregation. PGH2 via isomerases enzyme also produces prostaglandins D2, E2, and F2α [1,9].

### 3.2 NF-kB pathway

In the NF-κB signaling pathway, external stimuli such as cytokines, bacterial, and viral toxins initiate a cascade of molecular events [10]. This cascade begins when these stimuli bind to their receptors, activating the IκB kinase (IKK) complex comprised of IKKα, IKKβ, and the regulatory subunit IKKγ. The activated IKK complex phosphorylates the NF-κB inhibitor, IκB, marking it for degradation. This degradation allows the NF-κB complex to translocate into the nucleus, where it binds DNA and initiates the transcription of genes critical to inflammation and immune response, including cytokines like TNF-α, IL-6, IL-8, enzymes such as COX-2, iNOS, and cell cycle regulators including c-MYC and cyclin D1. This pathway's regulation of over 150 genes underlines its pivotal role in inflammation and its potential as a therapeutic target [11,12].

### 3.3 Mitogen-activated protein kinase (MAPK) pathway

MAPK (Mitogen-Activated Protein Kinase) pathway is the series of signaling events triggered by external stimuli leading to cellular responses. MAPK belongs to a class of serine/threonine protein kinases, which involves three concurrently activated protein kinases namely MAPKKK, MAPKK, and MAPK. Stimuli-activated MAPKKK goes on to phosphorylate MAPKK which in turn activates MAPK by dual phosphorylation [13]. MAPK can be grouped into three classes, extracellular signal-regulated kinase (ERK), c-Jun N- terminal kinase (JNK), and p38 MAPK.

ERK is divided into two classes ERK1 and ERK2, which respond to growth factors and mitogens to bring about cell differentiation. JNK is divided into JNK1, JNK 2 and JNK3. They activate c-Jun, AP-1 (Activator protein-1), and ATF-2 (Activating transcription factor-2) and are activated by exogenous factors like radiations, heat, stress, as well as inflammatory cytokines and growth factors [14]. p38 is activated by environmental stresses and inflammatory cytokines which lead to cell differentiation and apoptosis, they mediate anti or pro-inflammatory processes using transcription factors CREB (c-AMP response element-building protein) and ATF-2 [13].

### 3.4 JAK/STAT pathway

Cytokines activate the enzymes by engaging with receptors in the cytoplasm, by phosphorylating tyrosines on the intracellular domains of the receptors activating signal transducer activators of transcription (STATs) and Janus kinase (JAK). This is followed by the formation of STAT dimer that attaches to regulatory sites of genes in the nucleus, encoding cell proliferation and inflammatory proteins [15]. STAT pathway is also necessary for the retention of NF-kB in the nucleus and sustaining its activity [16].

### 3.5 Phosphatidylinositol-3-kinase (PI3K)/Ak strain transforming (Akt) pathway

The PI3K/Akt pathway plays a crucial role in the pathophysiology of immune-mediated disorders. Activation occurs when a ligand binds to a tyrosine kinase receptor, leading to the activation of phosphatidylinositol-(3,4,5)-trisphosphate (PIP3), which in turn activates Protein kinase B (PKB) or Akt. This activation is instrumental in the subsequent activation of the NF-κB transcription factor [17].

### 3.6 Other pathways

l-arginine in the presence of an electron donor and molecular oxygen produces Nitric oxide (NO), a free radical with a brief lifespan. Nitric oxide synthases (NOS) are a class of enzymes that catalyze the generation of NO in the body [18]. There are three definite subclasses of these enzymes which include endothelial NOS (eNOS), neuronal NOS (nNOS), and inducible NOS (iNOS). They are stimulated by external pathogens and pro-inflammatory cytokines, tumor necrosis factor (TNF), transcriptional factor, nuclear factor-kB (NF-kB), mitogen-activated protein kinase (MAPK), and Janus kinase (JAK) [9,18].

## 4 Anti-inflammatory potential of bioactive compounds

A vast range of anti-inflammatory traditional herbal drugs has found their way into modern therapy and becoming rapidly popular as a result of their low risk [1,19]. Because of the considerable detrimental effects of non-steroidal and steroidal drugs, they have been substituted with natural bioactive chemicals in nutritional supplements and herbal therapies [19].

The first step to determine a plant's biological activity is by preparing a plant extract with a suitable solvent, depending on their polarity, followed by phytochemical screening, purification, isolation, and finally characterization of the phytochemicals isolated [20].

Phytochemicals usually have physiochemical disadvantages like poor permeability, low bioavailability, low solubility, chemical instability under physiological conditions, and non-targeting of active sites, which lowers their therapeutic index and clinical effectiveness [21]. As a result of this impediment, scientists are working on better targeting drug release to optimize therapeutic impact and patient acceptance. Novel drug delivery methods have been developed to administer Phyto formulations. Some examples are liposomes, pyrosomes, nanosomes, transferases, ethosomes, microemulsions, microspheres, polymeric nanoparticles, solid lipid nanoparticles, and chitosan-based nanodrug delivery [22].

**Fig. 1:**
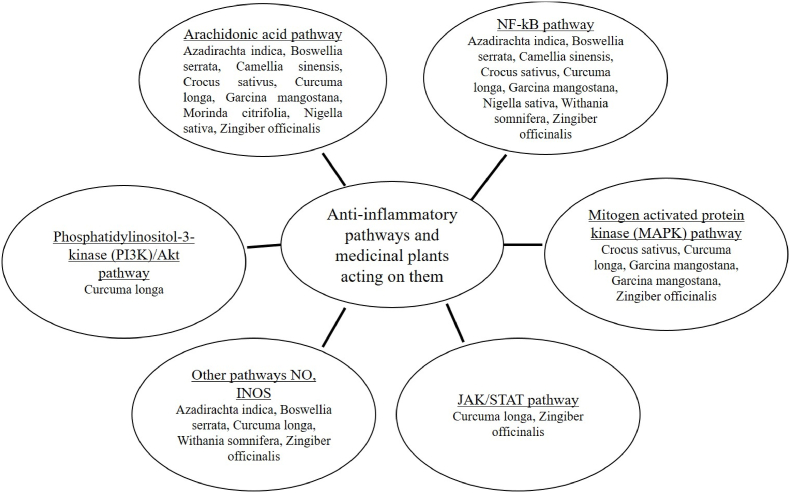
Schematic diagram enumerating traditional medicinal plants and their targets for anti-inflammatory response. NFkB: Nuclear factor kappa-B, JAK/STAT: Janus kinase/Signal transducers and activators of transcription, Akt: Ak strain transforming, NO: Nitric oxide, iNOS: inducible Nitric oxide synthase.

Plants containing flavonoids, phenolics, alkaloids, resins, essential oils, lignans, terpenoids, fatty acids, glycosides, steroids, and cannabinoids are common anti-inflammatory herbs whose impact has been studied in several ways [7,[23], [24], [25], [26], [27]]. Fig. 1 depicts a few medicinal plants and the anti-inflammatory mechanisms they use to lessen inflammation in various disorders. They may interact with proteins, inhibit enzymes like phospholipase A2, lipoxygenase, cyclooxygenase, and iNOS, and reduces pro-inflammatory factors such as prostaglandins, interleukins, nitric oxide, tumor necrosis factors, nuclear factor, mitogen-activated protein kinase, Janus kinase in the anti-inflammatory pathway [28]. Table 1 showcases the results gathered for ten traditional Indian medicinal herbs employed in the management of inflammatory disorders. The table includes details such as the specific parts of the plants used in treatment, their active phytochemical constituents, and the roles and targets of these constituents in the context of anti-inflammatory activity.

**Table 1:** An overview of commonly used medicinal herbs and their targets.

S. No	Plant	Phytoconstituent	Target	Model	Ref
1.	*Azadirachta indica*	Triterpenes: limolidesviz. Nimbolide, nimbin, nimbidin, azadiradione, azadirone, azadirachtin and Margosin, margosic acid	IL-1, IL-6, NO, TNF-α, COX-2, PGE2, glutathione peroxidase, glutathione reductase and glutathione-S- transferase	Arthritis, psoriasis, wounds, burns	[29,30,[33], [34], [35]].
2.	*Boswellia serrata*	Boswellia acids: 3-*O*-acetyl-11-keto-β boswellic acid, 11-keto-β-boswellic acid, α and β- boswellic acid	TNF- α, NF-kB, NO, iNOS, PGE2 LOX, COX	Arthritis, eczema	[[36], [37], [38], [39],^42^].
3.	*Camellia sinensis*	Polyphenols: catechin, epicatechin, epigallocatechin, epicatechin gallate, gallocatechin gallate, epigallocatechin gallate	IL-6, IL-8, NF-kB, TNF- α, COX-2	Arthritis, intestinal colitis	[45,47,51,52].
4.	*Crocus sativa*	Apocarotenoids: crocin, crocetin, picrocrocin, sarfanal	IL-4, nitrite, NO, COX 1 and 2, Th1/Th2 balance	Arthritis, bronchial asthma, hepatotoxicity,	[53,56,62].
5.	*Curcumin longa*	Curcuminoid: curcumin	IL-6, IL-1β, NF-kB, TNF- α, iNOS COX-2, PGE2	Arthritis, wounds, atopic dermatitis, psoriasis, periodontitis, cystic fibrosis, IBD, lung injury	[64,67,68,70].
6.	*Gracina mangostana*	Xanthones: α,β,γ- mangostins, gracinone B, gartanin, exuxanthone	H1 and H2 receptors, serotonergic receptors, NF-kB, iNOS, 12-LOX, MPO,Tlr-2 Icam-1, Vcam-1	Colitis, atopic dermatitis	[76,[79], [80], [81]].
7.	*Morinda citrifolia*	Iridoids: americaninA, asperuloside, narcissoside, asperulosidicacid, deacetylasperulosidic acid, Others: Rutin, quercetin vanillic acid, vanillin,borreriagenin, citrifolinin B epimer a and b, cytidine	Th1, Th2, IL-1β, TNF-α,IL-4, IL-5, IL-13, IL-31, IL-33, TNF-α IL-1β, TSLP TARC, skin protein barriers	Arthritis, asthma, allergy, atopic dermatitis	[83,84,[87], [88], [89]].
8.	*Nigella sativa*	Nigellone, α, β-pinene, carvacol, *p*-cymene, α-thujene, longifolene	TNF-α, IFN- γ,IL-1β, IL-6, PGE2, COX, 5-LOX	Bronchial asthma, atopic dermatitis, allergic rhinitis, liver function	[[90], [91], [92]].
9.	*Withania somnifera*	Steroidal alkaloids: withaferin A, steroidal lactones: whitanolide D, sitoindosides VII-X	Autoantibodies, IL-6, TNF-α, NF-kB	Arthritis, osteoarthritis, IBD, dermatitis, Parkinson's disease	[[98], [99], [100], [101], [102], [103],^105^].
10.	*Zingiber officinalis*	Volatile oils: Farnesene and zingeberene, bisabolene, β-sesquiphellandrene, 1,8- cineol, borneol, neral, geraniol, zingeberol	IL-4, IL-5, IL-13, TNF- α,NF-kB, JNK, COX-2, NO, iNOS, PGE2 inhibition	Hepatoma, arthritis, lung inflammation	[[110], [111], [112], [113]].

### 4.1 *Azadirachta indica*

Locally also called Neem, is rich in flavonoids, isoprenoids, saponins, and alkaloids [29].Most biologically significant is a class of triterpenes called limolides, which includes nimbolide, nimbin, nimbidin, azadiradione, azadirone, and azadirachtin. Neem extract has potent anti-inflammatory qualities, which have been used in the treatment of psoriasis and skin cancer [30]. It also demonstrated collagen-building properties that maintain skin elasticity and reverse aging, in addition to strong wound-healing properties [31]. Azadirachtin and nimbolide have reported significant antioxidant activity by neutralizing free radicals [32]. Azadiradione, a compound derived from the skin of *A.indica* reported potent analgesic and anti-arthritic activity by inhibiting arachidonic acid metabolism [33]. Another bioactive molecule identified, nimbidin, is claimed to inhibit neutrophils and macrophages in rats when given orally. It reduced inflammation by inhibiting the formation of PGE2 and decreasing the cytokine IL-1 [34]. The synergistic action of numerous phytochemicals present in the herb is assumed to be responsible for its biological activity; they repress COX2, inhibit IL-6 and TNF-α, and decrease NO by levels at the inflammation site [35].

### 4.2 *Boswellia serrata*

Widely known as Indian frankincense, Indian olibanum, or salai guggul in the Ayurvedic system. It contains terpenoids, essential oils, phenolics, and saponins. In various species of *Boswellia*, biological activity is primarily due to pentacyclic Boswellia acids, namely 3-*O*-acetyl-11-keto-β boswellic acid (AKBA), α- and β-Boswellia acids, and 11-keto-β-boswellic acid [36]. The oleo gum resin obtained has strong anti-inflammatory and anti-cancer properties. Several *in vitro* and *in vivo* studies have provided sufficient data to back up clinical trials [37]. *B.serrata* carries out anti-inflammatory activity through various mechanisms including inhibiting LOX, and COX and repressing oxidative stress. In a particular clinical study, a novel phytosome formulation of boswellic acids called Bosexil was used to treat psoriasis and erythematous eczema in patients. It's seen that boswellic acids significantly reduced scaling and erythema [38]. It has already been established that boswellic acids inhibit leukotriene production via 5- LOX [39].Boswellic acids also block the enzyme human leukocyte elastase which is generated during inflammation and hypersensitivity.AKBA inhibits NF-kB and suppresses TNF-α by inhibiting I kappa β kinase (IKK) thus being effective in treating psoriasis [40]. This traditional herb is also beneficial in the management of acute ulcerative colitis [41]. Phytoconstituents of *B.serrata* inhibit production of ROS [42]. There was a significant reduction in NO and lipid peroxidation levels as well as iNOS expression, hence drastically reducing inflammation in the mucosal and submucosal layers [41]. Boswellic acid extracts have also been shown to improve osteoarthritis of the knee in patients [43]. Boswellic acid has prominent systemic and topical anti-inflammatory activity. The effects of *B. serrata* pretreatment on LPS-induced cardiac injury in H9c2 cells was examined, and it was found that TNF-α, PGE2, IL-1, COX-2, iNOS, IL-6, and NO production were all reduced after pretreatment with *B. serrata* at different dosages (5, 15, and 45 g/ml) over 24 h [44].

### 4.3 *Camellia sinensis*

The leaves of *C.sinensis* are popular in making different kinds of black, green, and oolong tea. Green tea in particular is made from the unfermented leaves of the plant and contains the highest levels of polyphenols, thus making it the most biologically beneficial [45]. The six key catechins are catechin, epicatechin, epigallocatechin(EGC), epicatechin gallate (ECG), gallocatechin gallate, and epigallocatechin gallate (EGCG) which is the most researched and abundant of them all [46]. *In vitro*, studies outlined the effects of *C.sinensis* extract on LPS-induced macrophages. It demonstrated an improvement in colitic symptoms through the inactivation of NF-kB, IL-6, and IL-8 Y [47]. Oolong tea ethanol extract (OTEE), from *Camellia sinensis* and epigallocatechin gallate (EGCG) were tested for their anti-inflammatory effects on the lipopolysaccharide-induced murine macrophage cell line (RAW 264.7), and it was discovered that both have the potential to be used as anti-inflammatory medications due to their capacity to lower NO, COX-2, IL-6, IL-1, and TNF-α production in active macrophages [48]. An *in vivo* study examined the anti-inflammatory properties of hot water extract of tea (*Camellia sinensis*) flowers. It was discovered that in mice with immunological liver inflammation treated with tea flower extract (TFE), the levels of nitric oxide (NO), tumor necrosis factor-α (TNF-α), and interleukin-1β (IL-1β) were markedly suppressed [49]. Catechin-rich extracts were used to treat E. coli-induced intestinal colitis. Catechins effectively form hydrogen bonds with lipids in bacterial cell walls, leading to their rupture. It was also observed that the catechin-rich extract increased Anex1 (glucocorticoid-induced protein) expression, which suppressed phospholipase A2, associated with lower levels of inflammatory mediators, COX-2, TNF-α, and iNOS. The study further demonstrated that treatment with green tea extract significantly enhanced the expression of proliferation-related proteins, including PCNA (Proliferating Cell Nuclear Antigen) and CD44, and also restored Ki-67 expression in the damaged area, indicating the extract's ability to promote tissue repair and regeneration by stimulating cellular proliferation mechanisms [50]. A clinical experiment outlined the shielding effects of green tea in the early stages of chronic gastritis by inhibiting the conversion of nitrate to nitrite due to the polyphenols, that bring about anti-oxidant activity [51]. A study carried out by Clair Adcocks and the team detailed the anti-arthritic effects of green tea in human osteoarthritic and rheumatoid arthritis. Both EGCG and ECG successfully blocked the TNF- α and IL-1α thereby preventing deterioration of proteoglycan and type II collagen in cartilage [52].

### 4.4 *Crocus sativus*

*Crocus sativa* widely known as saffron contains several classes of phytoconstituents such as flavonoids, carotenoids, anthocyanins, glycosides, monoterpenes, and vitamins [53].

Research has exhibited that saffron shows strong anti-oxidant and anti-inflammatory properties. Aqueous and ethanolic extracts of *C.sativus* stigma show anti-inflammatory activity in experimental arthritic models [54]. *In vitro*, the anti-inflammatory activity of Crocin showed the blocking of PEG2 production via inhibition of both COX-1 and COX-2. Crocin therapy was found to have restorative effects in arthritic conditions by reinstating systemic anti-oxidants and inhibiting detrimental enzymes like exoglycosidase and cathepsin D levels in joints [55]. IL-1β generates matrix metalloproteinase (MMPS), which triggers osteoarthritis, crocin has an inhibitory impact on MMP-1 and MMP-3 production, thereby promoting the healing of joints [56]. Treatment with an extract from *Crocus sativus* to Balb/c mice drastically lowered serum TNF-α levels, other proinflammatory immune cell subtypes, and joint histological markers of osteoarthritis thus improving the disease progression [57]. Hydroalcoholic extract of the flower stigma was used to treat bronchial asthma, it showed a considerable decrease in lymphocytes, neutrophils, and eosinophils in lung lavage [58]. A recent review highlighted the multifaceted interactions between saffron (*Crocus sativus*) petals and their primary constituents, notably crocin, with the Nrf2 and NF-κB signaling pathways. Crocin enhances Nrf2 and reduces NF-κB expression, offering antioxidant and anti-inflammatory benefits. Saffron petals and their main constituents may have protective effects in numerous organs such as the brain, heart, and liver, via the Nrf2/heme oxygenase-1 (HO-1)/Kelch-like ECH-associated protein 1 (Keap1) signaling cascade contributing to its broad therapeutic impact. Such effects, supported by clinical evidence, underline crocin's potential in conditions like COPD, highlighting its comprehensive therapeutic capabilities across various conditions [59]. When serum cytokine and NO levels were analyzed, it revealed that hydroalcoholic extract tends to decrease IL-4, nitrite, and NO levels, but increased interferon- γ (IFN-γ). This difference between IL-4 and IFN-α implies that it has a stimulating effect on Th1 cells and an inhibitory effect on Th2 cells. Therefore an increase in Th1/Th2 balance, and anti-oxidant qualities are probable mechanisms pointing to asthma prevention [60]. The most recent finding examined the preventative effects of crocin supplementation on blood levels of IL-6 and TNF-α, exercise ability, and pulmonary function tests (PFT) in Chronic obstructive pulmonary disease (COPD) patients. It was discovered that crocin therapy dramatically improved exercise capacity and PFT in patients with COPD by lowering serum levels of inflammatory markers. It also enhanced IL6 levels and decreased TNF-α levels [61]. Another recent study showed the protective effect of C. *sativa* in methotrexate-induced hepatotoxicity during chemotherapy, by decreasing lipid peroxidation and NO circulation [62].

### 4.5 *Curcuma longa*

Popularly known as turmeric, has a long history of use in traditional medicine. It contains bioactive compounds called curcuminoids. Curcumin is the major curcuminoid and gives turmeric its vivid yellow color. Numerous *in vitro*, pre-clinical, and clinical research have been conducted to determine the safety and efficacy of its anti-inflammatory qualities [63]. Many studies have been conducted that show curcumin extract is effective in acute/chronic models of inflammation [64]. Mechanism of action can be attributed to the inhibition of IL-6 and IL-8 and other pro-inflammatory cytokines demonstrated *in vitro* studies [65]. Early investigations have shown that curcumin is an effective drug in wound healing, fresh juices of the rhizome were widely applied to wounds and aberrations on topical skin. Now it has been proven that it acts by lowering the production of prostaglandins and inhibiting lipoxygenase enzyme, thus reducing leukotriene formation [66]. It is known to suppress inflammation by repressing NF-kB and its activators [19]. Other vitamins and proteins in turmeric imitate fibroblastic action by the formation of collagen fibers [31]. A novel formulation of emu oil-based curcumin nanogel, administered topically has shown significant anti-inflammatory and analgesic activity in arthritis treatment. The mechanism of action was said to be the successful inhibition of IKK-α, COX, and iNOS [67]. Studies have also reported that curcumin modulates target receptors to mediate transcription factors like MAP kinase and AP-1 and curbs cytokine IL-1β generated matrix metalloproteinase (MMPS), MMP-1 and MMP-3, therefore assisting cartilage restoration at the joint [68]. The phytochemical has also been shown to have potent anti-ulcerogenic effects. *In vivo*, studies have shown that curcumin acts by decreasing MPO activity, TNF-α, and nitric oxide in ulcerative colitis. It also inhibits COX-2 and iNOS activity [69]. A study has shown, a mono-carbonyl analog of curcumin extract is used to treat liposaccharide (LPS) induced acute lung injury (ALI). It acts by down-regulating pro-inflammatory cytokines TNF-α, IL-6, IL-1β, and attenuated mRNA expression of COX 2 [70]. In a similar study, curcumin showed protective effects against acute lung injury (ALI) by upregulating Sirtuin 1 (SIRT1) and inhibiting NOD-like receptor protein 3 (NLRP3) inflammasome activation, thereby reducing inflammation and pyroptosis [71].Systemically administered curcumin produced a dose-dependent inhibition of NF-kB in gingival tissues of periodontitis disease-induced rats. It exhibited the inhibition of IL-6, TNF-α, and PGE2 by modulating the NF-kB pathway [68]. Curcumin has shown effectiveness in the treatment of cystic fibrosis. Oral administration of curcumin encapsulated in poly lactic co glycolic acid (PLGA) nanoparticles to increase its bioavailability, was utilized to treat two different cystic fibrosis strains in animal models, in an attempt to remedy the deficiencies associated with the ailment [72]. Reports have suggested anti-psoriatic effects of curcumin are by regulation of phosphorylase kinase activity, affecting calcium-dependent signaling pathways. In a recent study, LPS-induced IL-6 and TNF-α production in the murine macrophage cell line RAW264.7 was reduced by the *Curcuma longa* derivative bisacurone. Additionally, Bisacurone treatment of splenocytes from high-fat diet (HFD) mice decreased the generation of pro-inflammatory cytokines IL-6 and TNF-α following activation with TLR1/2 ligand Pam3CSK4 and TLR4 ligand lipopolysaccharide (LPS) [73]. The benefits of *Curcuma longa* essential oil (CLE) against the Toxoplasma gondii RH strain were studied both *in vitro* and *in vivo*, and it was discovered that it dramatically reduced the parasite by stimulating the immune system and lowering inflammation and oxidative stress [74]. Ongoing studies have shown that curcumin is used in the treatment of neurodegenerative diseases like Alzheimer's, Parkinson's, and Hodgkin's diseases [75].

### 4.6 *Garcinia mangostana*

Mangosteen as it is commonly referred to as a tropical plant is famous for its sweet-tasting fruit. The pericarp of the fruit has exhibited antioxidant, anti-allergic, anti-inflammatory, anti-viral, and anti-bacterial properties [76]. It was seen that α mangostin and its synthetic derivatives selectively block H1 and H2 receptors as well as serotonergic receptors [77]. *In vitro*, studies showed that α mangostin inhibited COX-2 while α and γ mangostin curtailed activation of iNOS in a dose-dependent manner [78].Another report suggested that α mangostin was more potent than γ mangostin and also inhibited the 12-LOX enzyme [49]. *In vivo*, experiments revealed α and γ mangostin extracted from fruit was a potent inhibitor of NO production and PEG2 metabolism via directly binding to active COX-2 enzyme. It was also seen to attenuate the gene expression of pro-inflammatory enzymes IL-6, IL-8 IL-1β, and TNF- α hence blocking the MAP kinase pathway [79].

Gracinone B, another bioactive phytoconstituent isolated, also had an inhibitory effect on PEG2 production and inhibited NF-kB activation [80]. Ethanolic extract of fruit pericarp containing α mangostin demonstrated protective effects against ulcerative colitis. It displayed anti-oxidant activity by activating catalase and superoxide dismutase that neutralized free radicals in serum and diminished myeloperoxidase (MPO) levels. Both extract and isolate suppressed TNF-α and toll-like receptor 2 (Tlr-2) gene expression. They also attenuated levels of adhesion molecules (Icam-1, Vcam-1), and monocyte chemoattractant protein (Mcp1) which led to the restoration of the mucosal layer and improvements in symptoms [81].Ethanolic extract was utilized to treat and prevent early stages of atopic dermatitis by downregulation of the NF-kB pathway and inhibition of IgE release from B cells and mast cell activation resulting in symptomatic relief [82].

### 4.7 *Morinda citrifolia*

Widely known as Noni, Indian mulberry or cheese fruit because of its pungent odor. Noni consists of flavonoids such as rutin, quercetin, isoquercitrin, kaempferol rutinoside, coumarins like scopoletin, esculetin, and is rich in iridoid glycosides, americanin A, asperuloside, narcissoside, asperulosidic acid, and deacetyl asperulosidic acid [83]. Other constituents present were vanillic acid, vanillin, borreriagenin, citrifolinin B epimer a and b, and cytidine. Americannin A was found to have the greatest anti-oxidant and anti-inflammatory activity.

Anti-allergic properties of ethanolic extracts of fruits and leaves of *M. citrifolia* outlined its inhibitory effects on IL-1β and TNF-α in *dinitrofluorobenzene* (DNFB) induced allergy in mice ear, thereby reducing ear swelling. The extracts had a prohibiting degranulation effect on RBL-2H3 cells in an *in vitro* β-hexosaminidase release assay [85]. In lipopolysaccharide-stimulated RAW264 cells, the combination of cannabidiol and *M. citrifolia* seed extract demonstrated more effective suppression of inducible nitric oxide synthase expression along with suppression of nitric oxide generation than cannabidiol therapy alone [86]. A pre-clinical study chronicled the effects of fermented Noni extract when administered to *dinitrochlorobenzene* (DNCB) induced atopic dermatitis in mice. It brought down immunoglobulin levels in a dose-dependent manner by targeting Th2 and Th1-mediated IG2a and IgE, IgG1 respectively. The extract attenuated Th2-mediated cytokine IL-4, IL-5, IL-13, IL-31, IL-33, and cytokine histamine, thymic stromal lymphopoietin (TSLP), and Thymus activation regulated chemokine (TARC) levels in serum. It was also seen to restore skin barrier proteins like filaggrin (FLG), loricrin (LOR), involucrin (IVL), zonula occludens-1 (ZO-1), and occludin (OCC) [87]. Alcoholic extract of Noni administered to arthritis-induced mice was seen to reduce MMP-9 levels in serum thus protecting cartilage and tissue from degradation [88]. Noni seed oil when clinically evaluated by repeat insult patch test on volunteers, showed inhibition of both COX-2 and 5-LOX [89]. An investigation has revealed Noni juice to be effective in treating chronic pulmonary inflammation in ovalbumin (OVA) sensitized rats. The Bronchoalveolar Lavage (BAL) fluid showed a significant decrease in lymphocytes, macrophages, eosinophils, and neutrophils and curbed NO to bring down oxidative stress. *Ex-vivo* bioassay also revealed that Noni juice acted as a calcium channel blocker on the jejunum muscle of rabbits to bring about an anti-spasmodic activity [83].

### 4.8 *Nigella sativa*

It is frequently referred to as black cumin or black seed. Oil from these seeds is well known for its anti-inflammatory properties [90]. The therapeutic activity of the plant is mainly attributed to the phytoconstituents, α, β-pinene, carvacrol, *p*-cymene, α-thujene, longifolene, unsaturated fatty acids like linoleic acid, oleic acid, and most importantly thymoquinone and poly thymoquinone (nigellone) [2,91]. *N. sativa* was found to be effective in the early stages of arthritic inflammation by blocking cytokine IL-6, inflammatory biomarker C-reactive protein, and decreasing oxidative stress [92]. Clinical studies have shown that the fixed oil of the herb is an effective analgesic and relieves allergic diseases such as bronchial asthma, atopic dermatitis, and allergic rhinitis by repressing inflammatory mediators [93,94].Twenty RCTs have examined the impact of *Nigella sativa* (*N. sativa*) supplementation on inflammatory and oxidative stress biomarkers in the adult population, and they found that it could significantly lower levels of CRP (C-Reactive protein), TNF-α, MDA (Malondialdehyde), SOD (superoxide dismutase), GPx (glutathione peroxidase), and TAC (total antioxidant capacity) [95]. Biological activity can be credited to thymoquinone and other bioactive compounds that inhibit COX-2 and 5-LOX pathways via suppression of pro-inflammatory enzymes TNF-α, INF- γ, IL-1β, IL-6 hence impeding PGE2 expression and leukotriene metabolism. Blocking lipid peroxidation also lowered biochemical enzymes such as myeloperoxidase, and articular elastase at joints thus lowering inflammation [96]. *In vivo*, studies reported the bioactive compound Nigellone derived from thymoquinone is reported to have an inhibitory effect on the 5-lipooxygenase and block histamine release from mast cells.Previously documented findings have suggested that *N.sativa* oil affects liver function by altering its lipid profile which leads to changes in the lipid metabolism in animals and humans in a dose-dependent manner [97].

### 4.9 *Withania somnifera*

The root of *Withania somnifera* is commonly utilized in traditional medicines. This herb, also known as Ashwagandha, includes a high concentration of natural steroidal lactone and alkaloids known as withanolides, and withaferin respectively [98]. Withaferin A and withanolide D are the most physiologically active, relieving inflammatory disorders, reducing stress, reversing aging, and acting as a potent anti-oxidant by elevating levels of catalase, glutathione peroxidase, and superoxide dismutase [99]. In the management of gout, *in vitro* and *in vivo* studies have shown that orally administered ashwagandha root powder showed a decrease in lysosomal enzymes, phosphatase cathepsin D, β-glucuronidase, β-galactosidase in serum [100].

Studies also displayed a decrease in arthritic inflammation of joints when treated with an extract of *W. somnifera* root. It acts by suppressing autoantibodies like rheumatoid factor, C-reactive protein, and anti-collagen type II, anti-nuclear, and citrullinated peptide antibodies due to its free radical scavenging activity [101,102]. It has been reported that fatty acids like linolenic acid, palmitic acid, and oleic acid extracted from the seeds of the herb are used to treat atopic dermatitis and psoriasis by impeding the NF-kB pathway, and cytokines like IL-6, TNF-α, etc [103]. *W. somnifera* has been observed to stimulate type I IFN production, potentially modulating immune responses. It may reduce type 2 immune responses in certain allergic conditions, reflecting the complex role of type I IFNs in antiviral defense and inflammation. This suggests *W. somnifera* as a promising immunomodulatory agent, offering potential benefits similar to those of synthetic adjuvants but with fewer adverse effects. Direct comparisons, however, are necessary to fully ascertain its safety and efficacy [104]. The application of a gel made from the herb roots showed protective action against inflammatory bowel disease. It inhibited NF-kB expression and excessive NO levels, thus restoring the bowel mucosal layers [105]. *W. somnifera* root formulation has been given clinically to patients suffering from Parkinson's disease and has resulted in symptomatic improvements [106]. Hepatic injury or failure is usually fatal. However, it has been reported that treatment with *W. somnifera* repairs the organ by restoring antioxidants and decreasing serum bilirubin [107].

### 4.10 *Zingiber officinale*

Also known as ginger is a common household herb used in everyday cooking. Historically it has been used to treat a variety of disorders, including inflammatory conditions [108]. Ginger root capsules have also been proven to be beneficial for reducing oxidative stress, and discomfort, and improving mobility in acute inflammation [109]. Gingerol, paradol, and shogaol are the active phenolics reported. It also contains volatile oils, made up of sesquiterpenes hydrocarbons including farnesene, zingiberene, bisabolene, β-sesquiphellandrene, monoterpene hydrocarbons like 1,8- cineol, borneol, neral, geraniol, and sesquiterpene alcohol, zingeberol [110].

The anti-inflammatory activities of red ginger were investigated *in vitro* and *in vivo* and were found to act by blocking the COX and LOX pathways [111]. The *Z. officinale* extract has shown potent effects in the management of musculoskeletal disorders like Rheumatoid arthritis and osteoarthritis. It considerably reduces joint swelling by blocking the granulomatous process (Funk et al., 2016). The mechanism of action can be attributed to the synergistic effects of several components present in the extract [112]. The main mechanism is attributed to the blocking of PGE2 production [110]. Anti-inflammatory activity is also brought about by repressing NO by blocking iNOS. The key bioactive component contributing to these effects was found to be Shogaol and gingerdiols. COX-2 inhibitors like shogaol, paradol, and gingerol were found to suppress COX 1 activity too. Indicating that their impact is not selective [111]. *In vivo*, studies have also shown the herb used in the management of hepatocellular carcinoma by inhibiting the NF-Kβ and TNF α pathway [113]. 6-gingerol has demonstrated anti-tumorigenic and anti-oxidative properties, by influencing critical mediators like MAPK, TNF- α, NF-kB, JNK, and COX-2 [114]. Th2 cells are engaged in the mediations of inflammatory cytokines, which cause hypersensitivity in the airway and worsen lung inflammation. It's been reported that 6-shogaol and 6-gingerol restricted allergic responses by blocking cytokines like IL-4, IL-5, and IL-13 [50].

## 5 Transitioning from preclinical to clinical trials

Transitioning from preclinical studies to clinical trials in the realm of herbal medicine presents several challenges. Identifying key active molecules within complex herbal extracts is often a hurdle due to their intricate chemical nature. Additionally, the cost of conducting clinical trials for herbal medicines can be prohibitively high, often surpassing the financial capabilities of researchers focused on natural products. Developing effective dosage forms that ensure stability and bioavailability of natural compounds is another significant challenge. Natural compounds frequently face stability issues, and their characterization can be difficult due to the presence of multiple active components. Furthermore, the bioavailability of many herbal constituents is typically low, necessitating innovative formulation approaches to enhance their therapeutic efficacy. To address these challenges, several strategies can be implemented. Standardizing the preparation of herbal medicines through rigorous protocols and quality control measures is crucial. Employing advanced techniques such as omics technologies and high-throughput screening can facilitate the identification and characterization of bioactive compounds. Enhancing the stability and bioavailability of natural compounds through novel formulation approaches, such as encapsulation in nanoparticles or development of advanced drug delivery systems, can improve their therapeutic potential. Early engagement with regulatory bodies and strict adherence to their guidelines can streamline the transition from preclinical to clinical stages. Collaborative research efforts, securing funding, and initiating pilot clinical trials will provide essential data to design larger studies, ultimately bridging the gap between preclinical promise and clinical application.

## 6 Significance of the study

This review holds significance by bridging the knowledge gap between traditional Ayurvedic practices and modern medicine, particularly focusing on the anti-inflammatory properties of selected Indian herbs. It contributes valuable insights into the potential therapeutic applications of these plants in managing inflammatory disorders. By detailing the molecular mechanisms of action of these herbs, this study lays a foundation for future research to build upon. The eventual goal is to substantiate these findings through rigorous research and development, ultimately translating them into clinically effective therapeutics. This has potential implications for global health, given the prevalence of inflammatory diseases and the growing demand for natural, affordable, and effective treatment options. The study reinforces the relevance of traditional knowledge in modern drug discovery, underlining the untapped potential of phytotherapy in contributing to a more holistic, integrative approach to healthcare.

## 7 Conclusion

In conclusion, this review provides critical insights into the anti-inflammatory potential of selected traditional Indian herbs and their complex molecular mechanisms, emphasizing their crucial role in modulating inflammatory signaling pathways. A thorough examination of the active components in the Neem tree (*Azadirachta indica*), Salai guggul (*Boswellia serrata*), Green tea (*Camellia sinensis*), Saffron (*Crocus sativus*), Turmeric (*Curcuma longa*), Mangosteen (*Garcinia mangostana*), Indian mulberry (*Morinda citrifolia*), Black cumin (*Nigella sativa*), Ashwagandha (*Withania somnifera*), and Ginger (*Zingiber officinale*) sheds light on their anti-inflammatory attributes and their interactions with various molecular targets in inflammation signaling pathways. While these discoveries represent a significant step towards bridging the gap between Ayurvedic wisdom and modern medicinal practice, they also underscore the imperative for more comprehensive research to authenticate these findings and drive the development of potent, plant-based therapeutics for managing inflammatory disorders. The continued exploration of this promising field holds immense potential for enriching global healthcare options and advancing patient outcomes.

## Author contributions

Saumya Upadhyay: Software, Formal analysis, Data curation, Writing – original draft. Rajan swami: Writing – review & editing. Shweta Shrivastava: Visualization, Data curation, Investigation, Writing – review & editing. Manish Kumar Jeengar: Conceptualization, Methodology, Validation, Visualization, Investigation, Writing – original draft, Writing – review & editing, Supervision, Project administration, Funding acquisition.

## Declaration of generative AI in scientific writing

There was no use of AI or AI-assisted technology in the writing of this manuscript.

## Funding sources

This work was supported by the common institute funding from 10.13039/100009526Amrita Vishwa Vidyapeetham.

## Conflict of interest

The authors declare the following financial interests/personal relationships which may be considered as potential competing interests: Dr. Manish Kumar Jeengar reports financial support was provided by 10.13039/100009526Amrita Vishwa Vidyapeetham. The authors declare that they have no conflict of interest.
