# Supplementary material for: Molecular insights into anti-inflammatory activities of selected Indian herbs
Source: J Ayurveda Integr Med. 2025 Mar 27;16(2):101081. doi: 10.1016/j.jaim.2024.101081 (PMC11986983; doi:10.1016/j.jaim.2024.101081)
Supplement: Multimedia component 1 [file mmc1.docx]

| **S. No.** | **Herbal Medicine** | **Search Terms Used** |
| --- | --- | --- |
| 1. | Neem Tree (*Azadirachta indica*) | "Azadirachta indica", "neem", "anti-inflammatory", "molecular targets", "pathways", "*in vivo*", "*in vitro*" |
| 2. | Salai Guggul (*Boswellia serrata*) | "Boswellia serrata", "Salai guggul", "anti-inflammatory", "molecular mechanisms", "*in vivo*", "*in vitro*" |
| 3. | Green Tea (*Camellia sinensis*) | "Camellia sinensis", "green tea", "anti-inflammatory", "in vitro", "in vivo" |
| 4. | Saffron (*Crocus sativus*) | "Crocus sativus", "saffron", "anti-inflammatory effects", "clinical studies", "*in vivo*", "*in vitro*" |
|  | Turmeric (*Curcuma longa*) | "Curcuma longa", "turmeric", "anti-inflammatory pathways", "research", "*in vivo*", "*in vitro*" |
| 5. | Mangosteen (*Garcinia mangostana*) | "Garcinia mangostana", "mangosteen", "anti-inflammatory", "mechanisms" |
| 6. | Indian Mulberry (*Morinda citrifolia*) | "Morinda citrifolia", "Indian mulberry", "anti-inflammatory", "molecular targets", "*in vivo*", "*in vitro*" |
| 7. | Black Cumin (*Nigella sativa*) | "Nigella sativa", "black cumin", "anti-inflammatory", "*in vivo*", "*in vitro*", "clinical evidence" |
| 8. | Ashwagandha (*Withania somnifera*) | "Withania somnifera", "ashwagandha", "anti-inflammatory", "*in vivo*", "*in vitro*" |
| 9. | Ginger (*Zingiber officinale*) | "Zingiber officinale", "ginger", "anti-inflammatory", "pathways", "*in vivo*", "*in vitro*", "clinical" |
